# Brain Bases of Reading Fluency in Typical Reading and Impaired Fluency in Dyslexia

**DOI:** 10.1371/journal.pone.0100552

**Published:** 2014-07-24

**Authors:** Joanna A. Christodoulou, Stephanie N. Del Tufo, John Lymberis, Patricia K. Saxler, Satrajit S. Ghosh, Christina Triantafyllou, Susan Whitfield-Gabrieli, John D. E. Gabrieli

**Affiliations:** 1 McGovern Institute for Brain Research and Department of Brain and Cognitive Sciences, Massachusetts Institute of Technology, Cambridge, Massachusetts, United States of America; 2 MGH Institute of Health Professions, Boston, Massachusetts, United States of America; 3 Harvard Graduate School of Education, Cambridge, Massachusetts, United States of America; 4 Athinoula A. Martinos Imaging Center, McGovern Institute for Brain Research, Massachusetts Institute of Technology, Cambridge, Massachusetts, United States of America; University of British Columbia, Canada

## Abstract

Although the neural systems supporting single word reading are well studied, there are limited direct comparisons between typical and dyslexic readers of the neural correlates of reading fluency. Reading fluency deficits are a persistent behavioral marker of dyslexia into adulthood. The current study identified the neural correlates of fluent reading in typical and dyslexic adult readers, using sentences presented in a word-by-word format in which single words were presented sequentially at fixed rates. Sentences were presented at slow, medium, and fast rates, and participants were asked to decide whether each sentence did or did not make sense semantically. As presentation rates increased, participants became less accurate and slower at making judgments, with comprehension accuracy decreasing disproportionately for dyslexic readers. In-scanner performance on the sentence task correlated significantly with standardized clinical measures of both reading fluency and phonological awareness. Both typical readers and readers with dyslexia exhibited widespread, bilateral increases in activation that corresponded to increases in presentation rate. Typical readers exhibited significantly larger gains in activation as a function of faster presentation rates than readers with dyslexia in several areas, including left prefrontal and left superior temporal regions associated with semantic retrieval and semantic and phonological representations. Group differences were more extensive when behavioral differences between conditions were equated across groups. These findings suggest a brain basis for impaired reading fluency in dyslexia, specifically a failure of brain regions involved in semantic retrieval and semantic and phonological representations to become fully engaged for comprehension at rapid reading rates.

## Introduction

Reading fluency, the ability to read accurately and at a rate that enables comprehension [1], [2], is a cornerstone of skilled reading. Developmental dyslexia, defined as a specific learning disability with a neurological basis, manifests as difficulty in reading accurately or fluently at the single word level [3]. The ability to extract the meaning of text requires the coordination of multiple processing demands [4], and readers with dyslexia can struggle with reading comprehension due to impaired decoding and/or through a slow reading rate [5], [6]. Despite consensus that reading fluency is essential for efficient reading and that reading dysfluency is a severe problem encountered by adolescents and adults with a history of dyslexia, there is little direct evidence regarding the neural systems critical for reading fluency and disruptions of those neural systems in dyslexia. Our aim was to identify the neural systems associated with fluent reading in typical adult readers, and discover how those systems differed in adults with dyslexia.

Reading fluency deficits are persistent and widespread in both adolescents and adults with a history of dyslexia [7], [8]. In contrast to effective interventions focusing on phonological deficits [9]–[11], dysfluent reading is especially difficult to remediate [6], [12]–[14]. Particularly beyond elementary school, remediation attempts to boost reading fluency yield only minimal improvements for dyslexic readers [15]. Challenges with reading fluency are not restricted to readers of English, but rather play a prominent role across languages [8], [16]–[20].

The brain basis of single word reading has been the focus of many functional magnetic resonance imaging (fMRI) and magnetoencephalography (MEG) reading studies, but the brain basis of reading connected text has been investigated less often [21]–[24]. In typical readers, brain regions associated with sentence reading include greater left-hemisphere than right-hemisphere activation in the inferior frontal gyrus, posterior superior and middle temporal gyri, as well as left occipito-temporal cortex, bilateral occipital cortex, left cerebellar declive, and dorsolateral prefrontal cortex [25]. Studies using fMRI to examine the neural correlates of sentence reading indicate that semantic processing is associated with activation in the temporal lobes, greater on the left, and left inferior frontal gyrus (BA47) [25], [26], and that processing accelerated text presentation is associated with activation in left occipito-temporal cortex [27]. For typically developing readers, higher scores on single-word reading tests are associated with increased activation during sentence reading in left temporo-parietal and ventral occipito-temporal regions [28]. Studies comparing readers with dyslexia to typical readers on whole-sentence reading have found relative hypo-activation in bilateral parietal cortices [24] and left occipito-temporal gyrus [17], but hyper-activation in left inferior frontal gyrus [17]. A MEG study of silent passage reading with each word presented individually at a constant rate (700 ms) found that, compared to typical readers, dyslexic readers exhibited hypo-activation bilaterally in the temporo-parietal and occipital cortices [28].

To date, fMRI studies of reading fluency using sentence stimuli have not examined and compared directly fluency per se in typical and atypical reading development (i.e., by varying and comparing fluency demands). One indirect approach involved correlating out-of-scanner standardized scores on tests of reading fluency with brain activations measured during scanning on tasks that did not vary fluency demands (reading sentences versus noun strings) [29]. In typical readers, higher fluency scores were associated positively with activations in left occipitotemporal cortex (BA18) and negatively with activations in right superior temporal gyrus, left insula, and left cerebellum. In a study that varied presentation rate on a word-by-word sentence reading judgment task in Hebrew with slower and faster rates, activation differences based on rate comparisons were not reported. However, typical adult readers showed greater activation in left premotor, left anterior insula/inferior frontal gyrus, right anterior insula, left middle temporal gyrus, and bilateral extrastriate visual cortex for each rate compared to fixation [30]. Readers with dyslexia showed less activation than typical readers during the relatively fast rate condition (versus fixation) in the posterior right temporal regions. Although these imaging studies considered fluency and involved reading sentences, as opposed to isolated words, none of the studies directly examined brain systems underlying reading fluency in readers with and without dyslexia.

Impaired reading fluency could arise from several sources. First, dyslexia has often been associated with impaired phonological awareness, even before the onset of reading instruction, that is thought to slow single-word decoding and, in turn, connected text composed of single words [31]. Second, dyslexia has also been associated with impaired naming speed for lists of stimuli, even for nonverbal material [32], and such slowness in processing for a series of stimuli may slow the reading of a series of words that constitute a sentence. Third, dyslexia has been associated with other temporal processing impairments for both linguistic and non-linguistic stimuli, such as deficits in general auditory processing [33], speech-specific processing [34], rise-time discrimination [35], or auditory sampling at optimal frequencies for analyzing language sounds [36]. Slowed temporal processing could impede reading fluency.

The major impediment for direct identification of neural systems important for reading fluency is that the kinds of educational and clinical tests used to measure fluency are not easily translated for fMRI experimentation. Further, it is not obvious what task would serve as an informative baseline for fluent reading. Therefore, the current study investigated the neural correlates of reading fluency by parametrically varying the rate at which sentences were presented one word at a time that were read silently. Accuracy and speed of comprehension, as indicated by plausibility judgments, verified reading performance. There were three rates of word presentation that corresponded to typical silent reading rates in 3^rd^ or 4^th^ grades (150 words per minute (wpm) (*slow rate*)), in 8^th^ or 9^th^ grades (240 wpm) (*medium rate*), and surpassing typical college reader expectations (600 wpm) (*fast rate*).

We examined neural systems that may be important for fluency by identifying brain regions that changed activation in response to changes in presentation rate in typical young adult readers and readers with dyslexia. The use of three different reading rates allowed for a comparison of typical readers and readers with dyslexia with performance differences equated by examination of a faster rate in the typical readers compared to a slower rate in readers with dyslexia. Such performance-equated comparisons permit consideration of whether activation differences between typical readers and readers with dyslexia are simply a consequence of performance differences or are related more directly to the cause of reduced fluency in dyslexia [37]. We hypothesized that brain regions showing increasing activation with increasing rates of presentation would be important for reading fluency, and that activation patterns would differ in readers with and without dyslexia.

## Methods

### Ethics statement

Written informed consent for participation in the study, approved by the Massachusetts Institute of Technology (MIT) and Harvard University Institutional Review Boards, was obtained from all participants.

### Participants

Participants recruited from online recruitment in the local community of urban and suburban areas met inclusion criteria of: between 18–35 years of age; native English speakers; completion of high school or higher levels of formal education; right handedness as indicated by responses to a questionnaire adapted from the *Edinburgh Handedness Inventory* [38]; no contraindications to MRI; and absence of neurological or psychiatric impairments or associated medications. Participants completed a behavioral testing session at MIT and an MR scanning session at the Athinoula A. Martinos Imaging Center, McGovern Institute for Brain Research at MIT.

### Behavioral assessment

Standardized measures of cognitive, reading, and reading-related abilities were administered to participants by trained researchers at MIT. The testing battery included measures of cognitive ability, *Wechsler Abbreviated Scale of Intelligence, 3^rd^ Ed.* (WASI) [39]; phonological processing, *Comprehensive Test of Phonological Processing* (CTOPP) [40]; and rapid naming, “Letters,” “Numbers,” and “2-set” from the *Rapid Automatized Naming and Rapid Alternating Stimulus Tests* (RAN/RAS) [41]. Untimed reading ability was indexed by accuracy for reading real words and pseudowords, “Word Identification” and “Word Attack” from the *Woodcock Reading Mastery Test-Revised, NU* (WRMT) [42]. Timed reading ability was indexed by accuracy for reading real words and pseudowords within time limits, “Sight Word Efficiency” and “Phonemic Decoding Efficiency” from the *Test of Word Reading Efficiency* (TOWRE) [43]. Untimed and timed measures of connected text reading were used to index reading comprehension ability by using cloze sentences, which are sentences in which a reader is asked to supply a word that has been removed from a passage in order to assess comprehension. These measures were “Passage Comprehension” from the WRMT, text passages from the *Nelson-Denny Reading Test* (NDRT) [44], and sentences that required a semantic plausibility judgment within a time limit, “Reading Fluency” from the *Woodcock-Johnson III* (WJ) [45]. Reading rate was recorded as the number of words in a text passage read silently at a typical pace within a time limit, “Reading Rate,” NDRT. Participants completed a background questionnaire regarding developmental history of language and literacy skills.

### Participant groups

Two participant groups were included in this study. Typical readers (n = 12; 5 female) were between 18–28 years of age (*M* = 22.5; *SD*  = 3.1) and earned a score at or above the 25^th^ percentile on four measures of untimed or timed single word reading (TOWRE, WRMT; Table 1). Readers with dyslexia (n = 12; 8 female) were between 18–31 years of age (*M* = 23.3; *SD*  = 4.1) and had both a history and a clinical diagnosis of reading disability, and were also currently scoring below the 25^th^ percentile rank on at least two subtests of timed or untimed single word or pseudoword reading measures (TOWRE, WRMT; Table 1). All participants demonstrated cognitive performance at or above the expected mean range of 100±15 (WASI). The two groups did not differ significantly on age or nonverbal cognitive ability (Table 1). Among participants who reported ethnicity, 100% of adults in the control group reported having a Caucasian background. Among participants with dyslexia, 75% reported having a Caucasian background, one identified as Black, and 2 elected not to respond. There were no between group differences in education level attained.

**Table 1:** Participant Scores for Typical Readers and Readers with Dyslexia.

		Typical Reader	Dyslexic	p-values: Typical vs.
		Group	Group	Dyslexic
*N*		12	12	–
Age		22.5±3.1	23.3±4.1	.61
**Construct**	**Behavioral Measure**			
Cognitive Abilities	WASI – Verbal	121.17±13.04	109.00±7.31	.010
	WASI – Performance	114.67±8.2	110.58±6.05	.181
Phonological Processing	CTOPP – Elision	11.00±0.85	8.27±1.62	.0005
	CTOPP – Blending Words	11.00±1.41	8.83±2.76	.024
	CTOPP – Memory For Digits	12.33±2.71	12.27±1.49	.948
	CTOPP – Nonword Repetition	9.33±1.92	7.42±1.51	.013
Sublexical Fluenc	RAN – Numbers	112.83±5.28	105.00±6.84	.005
	RAN – Letters	112.00±3.24	102.00±7.39	.0005
	RAN – RAS	112.33±5.85	101.67±11.20	.008
Word Reading Accurac	WRMT – Word Identification	108.83±10.07	90.25±8.97	.0005
	WRMT – Word Attack	111.00±12.23	93.92±6.99	.0005
Word Reading Fluency	TOWRE – Sight Word Efficiency	103.58±8.24	85.00±6.36	.0005
	TOWRE – Phonemic Decoding Efficiency	100.17±6.83	80.83±8.94	.0005
Connected Text Reading Fluency	WJ – Reading Fluency	118.55±8.65	93.92±10.03	.0005
Connected Text Reading Comprehension	WRMT – Passage Comprehension	115.00±11.10	106.07±6.90	.031
	NDRT – Reading Comprehension	241.00±11.34	209.75±14.64	.0005
Connected Text Reading Rate	NDRT – Reading Rate	224.58±23.83	186.33±11.60	.0005
**In-Scanner Task Performance**				
Accuracy (% correct)	Fast	81±10	64±11	.001
	Medium	94±6	83±9	.002
	Slow	95±3	92±6	.150
Reaction Time (ms)	Fast	975.39±254.72	1230.13±312.70	.040
	Medium	629.88±187.12	954.92±259.49	.002
	Slow	512.65±218.92	755.73±168.58	.006

Mean ± SD; *p* values below .05 are statistically significant based on two-tailed t-tests. Note: Standard scores are based on a mean of 100 and a standard deviation of 15 (average range of 85-115) except for the CTOPP (based on mean of 10 and a standard deviation of 3; average range of 7-13) and NDRT (based on mean of 200 and a standard deviation of 25; average range of 175-225). SD  =  Standard deviation.

### Task design and materials

The sentence reading paradigm consisted of five words presented sequentially, followed by a question mark, for each trial. Participants were asked to indicate via button press whether each sentence was semantically plausible (e.g., *Bulls charge with great ferocity*) or semantically nonplausible (e.g., *Kangaroos type for their jobs*). Participants practiced the paradigm with unique stimuli prior to the scanning session to ensure understanding of task directions and mastery of task demands.

Words in each sentence trial were presented at one of three speeds: Slow (400 milliseconds/word), Medium (250 milliseconds/word), or Fast (100 milliseconds/word). The slow sentence presentation rate, corresponding to 150 words per minute, was commensurate with a silent reading rate for typical readers in grade three or four [46], [47]. The medium sentence presentation rate, corresponding to 240 words per minute, was consistent with typical grade eight or nine silent reading rates [44], [46]. The fast sentence presentation rate, corresponding to a rate of 600 words per minute, was selected to be challenging for typical adult readers by surpassing the typical college-level silent reading rate of about 280 words per minute and efficient reading rates of about 500 words per minute [46], [47].

The nouns and verbs in sentence sets were matched for written frequency and number of syllables in three ways: between runs (run 1, run 2), between conditions (Slow, Medium, Fast), and between sentence types (plausible, nonplausible). One-way analysis of variance (ANOVA) was used to compare stimuli characteristics, which were compiled using the MRC Psycholinguistic Database (www.psy.uwa.edu.au/mrcdatabase/uwa_mrc.htm). First, sentences were balanced across run 1 and run 2 for written frequency [nouns: *F*(1, 295)  = 1.08, *p* = .30; verbs: *F*(1, 150)  = 1.30, *p* = .26] and for number of syllables [nouns: *F*(1, 318)  = 0.02, *p* = .90; verbs: *F*(1, 154)  = 1.65, *p* = .20]. Second, sentences were matched across the three conditions (Slow, Medium, Fast) for written frequency [nouns: *F*(2, 294)  = 1.27, *p* = .28; verbs: *F*(2, 149)  = 1.37, *p* = .26] and for number of syllables [nouns: *F*(2, 317)  = 0.94, *p* = .39; verbs: *F*(2, 153)  = 0.08, *p* = .93] to minimize effects of reading time that could be impacted by longer or less frequent words. Third, sentences were matched across the two types, plausible and nonplausible, for written frequency [nouns: *F*(1, 295)  = 0.90, *p* = .34; verbs: *F*(1, 150)  = 0.17, *p* = .68] and for number of syllables [nouns: *F*(1, 318)  = 2.35, *p* = .13; verbs: *F*(1, 154)  = 0.92, *p* = .34]. The sequence of sentence and rest trials was based on the output from a randomization program (OPTSEQ2) that generated three equivalent versions of the fluency task. Each version used the same sentence stimuli, but balanced the rate of presentation so that each sentence was presented in each condition across the three versions.

A jittered event-related design was used in which sentence conditions and a rest condition were randomly intermixed. The duration between trials, which constituted the rest condition, varied (i.e., was jittered). For the rest condition, participants were asked to stare at a fixation cross; this condition served as a low-level baseline. Rest trials were interspersed at random between sentence trials (i.e., jittered time periods), and the duration of the rest trials ranged from 200–2200 milliseconds at increments of 200 (e.g., 200 ms, 400 ms, 600 ms, … , 2200 ms). The total presentation time of each sentence condition was equal to the total duration of rest trials. Each sentence trial totaled four seconds and consisted of a sentence and a question mark. The question mark duration made up for the varying length of the sentence duration to total 4 seconds (i.e., 3500 ms for Fast, 2750 ms for Medium, and 2000 ms for Slow).

Each participant completed two consecutive runs (10.4 minutes each) of the sentence-reading fMRI task. Each run consisted of 78 sentences, with 13 semantically plausible and 13 nonplausible sentences at each of the three rates. Thus, there were 156 sentences in total across both runs. Stimuli were presented on a rear projection screen in white on a black background via PsychToolBox software [48]. The screen size, zoom, and focus were calibrated for each participant to ensure that the entire visual field of the projected images was visible through the mirror mounted on the head coil.

### Imaging data acquisition

Imaging was performed using a Siemens 3T MAGNETOM Trio, a Tim System, (Siemens Medical Solutions, Erlangen, Germany) and a commercial 12-Channel Matrix head coil (Siemens Medical Solutions, Erlangen, Germany). To minimize head movement, tetrahedron-shaped foam pads were placed between the head coil and either side of the participant's head. Sagittal localizer scans were aligned to a multi-subject atlas to derive automatic slice prescription for consistent head position across participants. At the beginning of each functional scan, five images (10 second duration) were discarded to allow for T1 equilibration. High-resolution structural whole-brain images were acquired using a T1-weighted anatomical scan (128 slices per slab; 256×256 matrix; 256 mm FOV; 1.33 mm slice thickness; 0.63 mm interslice gap; TR * = *2530 ms; TI  = 1100 ms; TE  = 3.39 ms; flip angle  = 7°).

Functional data were collected using a gradient echo T2*-weighted EPI sequence sensitive to the BOLD contrast. The gradient-echo EPI images were acquired with PACE [49], an online motion correction algorithm that minimizes movement-related artifacts by adjusting the system gradients and the acquisition field of view between one whole brain acquisition and another for participant movement. Thirty-two sagittal slices parallel to the anterior commissure-posterior commissure (AC-PC) line were imaged (voxel size of 3.1×3.1×4.0 mm, 64×64 mm matrix, 200 mm field-of-view, 4 mm slice thickness, 0.8 mm inter-slice gap). Other imaging acquisition parameters included: TR * = *2000 ms, TE  = 30 ms, flip angle  = 90°, bandwidth  = 2298 Hz/Px, echo spacing  = 0.5 ms.

### In-Scanner recording of performance

Accuracy and reaction time in judging semantic plausibility were recorded when participants responded via button press for each trial. The scanner paradigm was programmed to take one response from the time that the trial began (first word in the sentence) to the end of the trial (question mark).

### fMRI data analysis

The neural correlates associated with increasing reading fluency demands were measured using a within-subjects design and a parametric modulation analysis, which creates a statistical parametric mapping of the significance of the correlation between cognitive parameters and physiology [50]. Preprocessing and statistical analysis were performed using statistical parametric mapping software (SPM8; Wellcome Department of Cognitive Neurology, London, UK; http://www.fil.ion.ucl.ac.uk/spm). During preprocessing, data were realigned to the first functional volume and spatially normalized using the mean functional volume to the Montreal Neurological Institute (MNI) template. Normalized images were smoothed using a Gaussian filter (6-mm full width at half maximum) to decrease spatial noise.

Analysis included individual and group level statistics. For the individual level analysis, the stimuli (defined as the start of the first word and the end of the last word) were modeled as box-car functions aligned with the onset of each stimulus, the width of which corresponded to the duration of each stimulus. The expected BOLD responses to the stimuli were obtained by convolving a canonical hemodynamic response function with the modeled stimuli. A high-pass filter (cutoff  = 128 s) was used on both the data and the model to reduce impact of physiological noise. The mean voxel value was used for the global calculation. Grand mean scaling was based on session specific parameters. Global normalization was not used.

Outlier image volumes in the BOLD time series were identified based on either the mean intensity of image volume greater than 3 standard deviations from the mean intensity of the time series or the largest voxel movement of the image volume greater than .5 mm, based on scan-to-scan movement. Image volumes were masked by a binary image created from the functional time series (using the same procedure as that used to create the SPM analysis mask). Outlier images were included as nuisance regressors in the first-level analysis per person. The typical reader group (*M* = 11.7, *SD*  = 11.3) and the dyslexic group (*M* = 7.0, *SD*  = 5.5) did not differ in the number of outlier images (*t*(22)  = 1.27, *p* = .22).

A random effects model [51] was used to characterize group level effects (second-level analysis). Brain regions were identified using a threshold of *p*<.001 cluster-level FDR corrected for multiple comparisons and using a cluster extent threshold (ET) of 10 voxels or more. We used the Topological False Discovery Rate (FDR) calculations from SPM8. SPM8 computes FDR by assigning corrected *p*-values to the local maxima. Peak-wise FDR has fewer false positives than conventional voxel-wise FDR [52], [53].

The comparison between the typical reader group and the dyslexic group was based on differences between mean parameter estimates in a linear parametric modulation contrast (Fast > Medium > Slow) using a threshold of *p*<.01 cluster-level FDR corrected for multiple comparisons. An independent samples t-test was used to characterize clusters showing significant difference between groups. Within and between group comparisons were also completed for Medium > Slow to ensure that the parametric comparison was not driven by aberrant activations elicited by the fast rate, which exceeded expectations for even typical adult readers. For these analyses, all trials (correct and incorrect) were used to maximize power and not bias data-points in favor of the typical reader group, who answered more items correctly compared to readers with dyslexia. In addition, the Fast > Slow contrast for typical readers was compared to Medium > Slow for dyslexic readers because these contrasts yielded comparable behavioral performance between the groups. For this comparison, in-scanner accuracy changes in performance between presentation rates did not significantly differ between groups.

We also examined as an *a priori* region of interest the putative visual word form area (VWFA), which has been associated with rapid visual analysis of text for typical readers [54]. The ROI was defined as a 10 mm sphere with the location taken from the imaging literature (Standard Talairach Coordinates: x = −43, y = −54, z = −12; [55]).

## Results

### Behavioral measures

The typical reader group performed significantly better than the dyslexic group on standardized measures of verbal cognitive abilities, phonological processing (with the exception of Memory for Digits, an index of phonological memory), rapid naming (letters, numbers, 2-set), timed and untimed single word reading, timed and untimed text comprehension, and reading rate (Table 1; independent samples t-tests, two-tailed, all *p*<.05). The typical reader group and the dyslexic group did not differ significantly on non-verbal cognitive abilities (*p* = .18).

### Scanner task performance

In-scanner performance (Table 1) for the typical reader group and the dyslexic group was analyzed with a 3×2 repeated measures ANOVA, with Condition (Fast, Medium, Slow) as a within-subjects factor and Group (typical readers vs. readers with dyslexia) as a between-subjects factor. The typical reader group was more accurate than the dyslexic group as indicated by a significant main effect for Group [*F*(1,22)  = 14.16, *p*<.001]. Accuracy declined with greater rates of presentation as indicated by a significant main effect of Condition [*F*(2,21)  = 61.34, *p*<.0005]. Accuracy differences between groups varied as a function of presentation rate as shown by a significant Group X Condition interaction [*F*(2,21)  = 8.46, *p*<.002], with the dyslexic group performing significantly worse on Fast [*t*(22)  = 4.05, *p*<.001] and Medium [*t*(22)  = 3.59, *p*<.002] conditions, but not on the Slow condition [*t*(22)  = 1.49, *p* = .15]. Groups did not differ on rates of response across conditions [*t*(22)  = 1.77, *p* = .09], indicating that both groups of participants had sufficient time to respond to items.

The typical reader group was faster to respond than the dyslexic group as indicated by a significant main effect for Group [*F*(1,22)  = 9.76, *p*<.005]. Responses were slower as presentation rates increased as shown by a significant main effect of Condition [*F*(2,21)  = 49.06, *p*<.0001]. The Group X Condition interaction was not significant [*F*(2,21)  = 2.10, *p* = .15].

In-scanner performances for the typical reader group and the dyslexic group were compared to determine if performance was comparable at the Fast rate for the typical reader group and at the Medium rate for the dyslexic group. Independent samples t-tests showed that performance for the typical reader group during the Fast-rate condition did not differ significantly from performance for the dyslexic group during the Medium-rate condition for accuracy [*t*(22)  = 1.04, *p* = .31] or reaction time [*t*(22)  = 0.49, *p* = .63]. Thus, we included these conditions as a performance-equated group comparison. Further analysis indicated that the difference between Slow and Fast conditions for the typical reader group and the difference between Slow and Medium conditions for the group with dyslexia was not statistically significant for accuracy [*t*(22)  = 1.58, *p* = .13], but was for reaction time, with the typical reader group exhibiting a larger difference than dyslexic group [*t*(22)  = 2.99, *p*<.05].

### Relation of in-scanner performance with standardized measures of reading fluency and phonological awareness

We examined the relationship between behavioral performance in the scanner and a standardized test of reading fluency on which the task was based (“Reading Fluency,” WJ) across participants in both groups using correlation analysis (uncorrected for multiple comparisons). There were significant negative correlations between age-standardized Reading Fluency scores and in-scanner reaction time at all three reading rates: Slow (standard scores: *r* = −.69, *p*<.01); Medium (standard scores: *r* = −.68, *p*<.01), and Fast (standard scores: *r* = −.54, *p*<.01). There were significant positive correlations between Reading Fluency scores and accuracy at the Medium and Fast rates, which did not show ceiling effects for accuracy: Medium (standard scores: *r* = .47, *p*<.05); Fast (standard scores: *r* = .63, *p*<.01). These correlations reflected group differences between the typical reader and dyslexic groups, because the correlations were not significant within either group alone.

We also examined the relationship between phonological processing and in-scanner performance. Standard scores from a measure of phonological awareness (“Elision,” CTOPP) showed significant correlations with in-scanner reaction time for Medium (*r* = −.49, *p*<.05) and Slow (*r* = −.43, *p*<.05) rates, and accuracy for Fast (*r* = .44, *p*<.05) and Medium (*r* = .47, *p*<.05) rates. Other measures of phonological awareness and phonological memory were not significantly correlated with in-scanner performance (*p*>.05). Once again, these correlations reflected group differences between the typical reader and dyslexic groups, because the correlations were not significant within either group alone.

### fMRI Activation for Typical Readers

#### Fast > Medium > Slow

**Figure 1:**
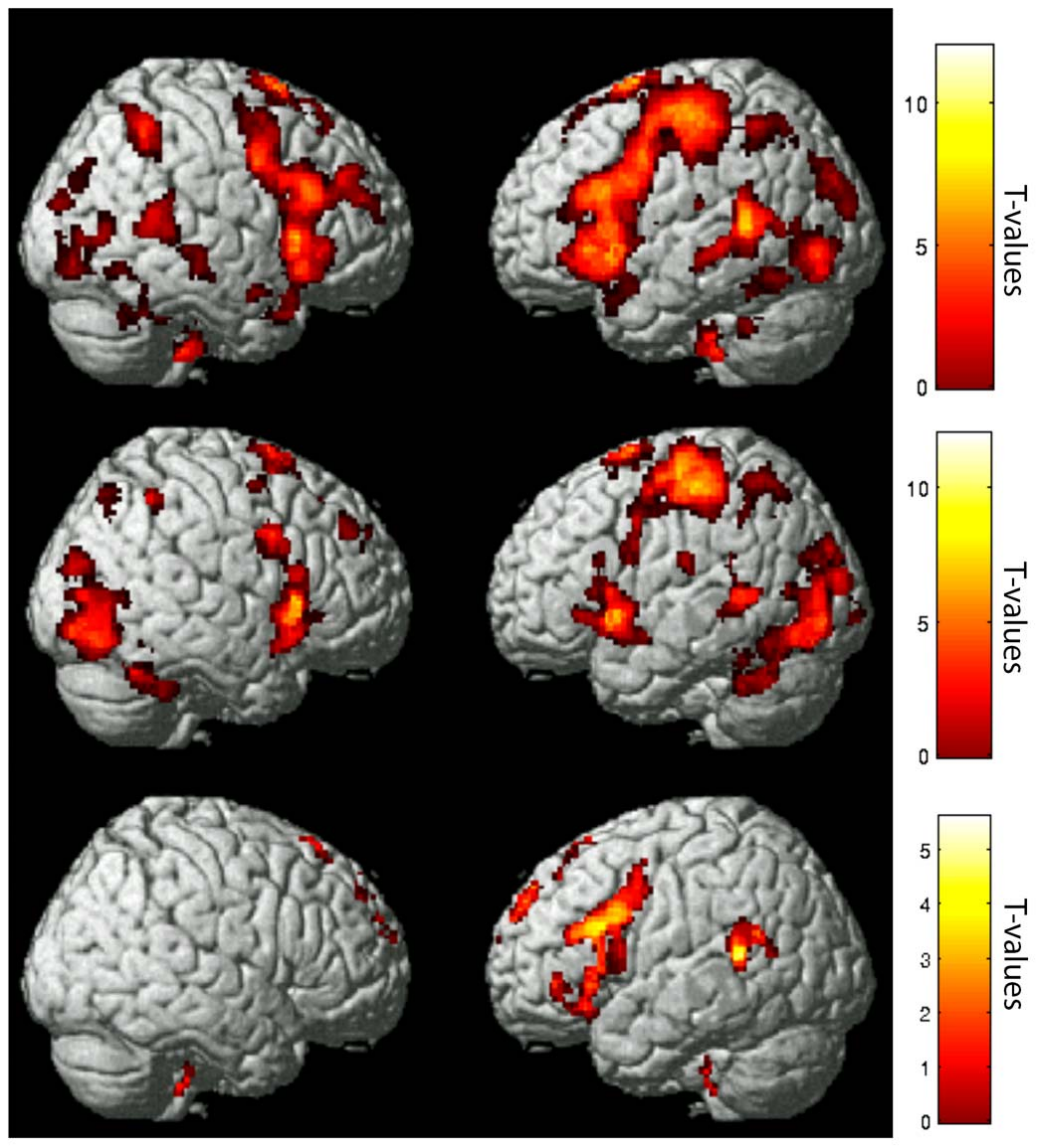
Sentence presentation rate differentially impacts brain activation by group. Fast > Medium > Slow parametric modulation (cluster level FDR corrected) for a) Typical Reader Group (p<.001) (top panel); b) Dyslexic Group (p<.001) (middle panel); c) Typical > Dyslexic Groups (p<.01) (bottom panel). Color bar indicates T-values.

Typical readers showed greater activation for faster rates of word presentation in a distributed cortical network including peak activations in left superior frontal gyrus, left middle temporal gyrus, right superior temporal and insular regions, left inferior occipital gyrus, right middle occipital gyrus, and cerebellar and subcortical regions (Table 2 & Figure 1, top panel).

**Table 2:** Activations for Fast > Medium > Slow contrasts for Typical Readers.

Region (peak activation)	BA	x	y	z	Cluster extent	*p*	Z score
***Fast > Medium > Slow***							
**Frontal Lobe**							
L. Superior frontal gyrus	6	−2	8	70	5008	<.0001	6.61
**Temporal Lobe**							
R. Insula	13	30	26	0	3841	<.0001	6.11
L. Middle temporal gyrus	21	−50	−46	8	1270	<.0001	5.53
R. Superior temporal gyrus	22	54	−46	14	666	<.0001	5.47
**Parietal Lobe**							
N/A							
**Occipital Lobe**							
L. Inferior occipital gyrus	19	−34	−76	−4	1082	<.0001	5.32
R. Middle occipital gyrus	19	36	−88	18	81	.017	4.52
R. Middle occipital gyrus	19	48	−78	4	327	<.0001	4.04
**Cerebellum**							
L. Cerebellum	N/A	−38	−46	−38	199	<.0001	4.42
L. Cerebellum	N/A	−8	−78	−38	83	.017	4.30
**Sub-lobar**							
Thalamus	N/A	−8	−16	4	16152	<.0001	6.22
R. Caudate	N/A	12	12	8	115	.006	4.11
Brainstem	N/A	4	−34	−46	577	<.0001	4.98
***Medium > Slow***							
**Frontal Lobe**							
L. Superior frontal gyrus	6	−2	8	60	2605	<.0001	6.42
R. Middle frontal gyrus	46	48	26	26	216	.001	5.24
R. Inferior frontal gyrus	47	34	32	−2	324	<.0001	4.43
R. Precentral gyrus	6	38	−4	38	90	.028	4.42
**Temporal Lobe**							
R. Middle temporal gyrus	22	50	−40	6	104	.021	4.39
**Parietal Lobe**							
L. Postcentral gyrus	3	−40	−24	48	6078	<.0001	5.98
R. Inferior parietal lobule	40	36	−46	50	132	.009	3.89
R. Precuneus	19	30	−68	36	87	.028	3.66
**Occipital Lobe**							
R. Lingual gyrus	18	18	−80	−2	6923	<.0001	5.46

*p*<.001, cluster level FDR corrected (T = 3.50); ET  = 10. N/A  =  Not applicable. L. =  Left hemisphere. R. =  Right hemisphere. Coordinates reported in Talairach space.

#### Medium > Slow

In typical readers, comparisons excluding the fastest rate continued to demonstrate robust activations in networks including frontal systems with peaks in the superior frontal gyrus that was situated medially and extending to both hemispheres; right middle and inferior frontal gyrus; and right precentral gyrus. Activations also included clusters with peaks in the right middle temporal gyrus, left postcentral gyrus extending to inferior frontal gyrus, right inferior parietal lobule and precuneus, and a cluster with a peak in right lingual gyrus that included bilateral fusiform gyri and left cuneus (Table 2).

### fMRI Activation for Dyslexic Readers

#### Fast > Medium > Slow

Dyslexic readers showed greater activation for faster rates of word presentation in a distributed cortical network including left superior frontal gyri, right middle frontal gyrus, bilateral insular and middle temporal regions, left postcentral gyrus, left superior parietal lobule, bilateral inferior parietal lobule, right precuneus, and cerebellar and subcortical regions (Table 3 and Figure 1, middle panel).

**Table 3:** Activations for Fast > Medium > Slow contrasts for Readers with Dyslexia.

Region (peak activation)	BA	x	y	z	Cluster extent	*p*	Z score
***Fast > Medium > Slow***							
**Frontal Lobe**							
L. Superior frontal gyrus	8	6	16	50	3902	<.0001	6.06
R. Middle frontal gyrus	9	36	44	36	94	.023	4.42
R. Middle frontal gyrus	6	28	4	46	84	.029	4.16
**Temporal Lobe**							
L. Insula	13	−36	12	2	1432	<.0001	5.93
R. Insula	13	30	24	2	1866	<.000	6.15
L. Middle temporal gyrus	21	−62	−46	8	324	<.0001	4.29
R. Middle temporal gyrus	21	48	−34	−4	69	.049	3.41
**Parietal Lobe**							
L. Postcentral gyrus	3	−36	−30	52	2626	<.0001	5.77
L. Superior parietal lobule	7	−30	−52	52	817	<.0001	4.65
L. Inferior parietal lobule	40	−54	−20	24	90	.025	3.75
R. Inferior parietal lobule	40	46	−50	52	96	.023	3.71
R. Precuneus	7	26	−68	50	223	.001	3.81
**Occipital Lobe**							
N/A							
**Cerebellum**							
Anterior lobe	N/A	0	−54	−30	216	.001	3.96
**Sub-lobar**							
L. Thalamus	N/A	−10	−20	0	11851	<.0001	5.93
Cingulate gyrus	23	8	−22	26	99	.023	4.53
***Medium > Slow***							
**Frontal Lobe**							
R. Superior frontal gyrus	8	6	16	52	1744	<.0001	6.15
R. Middle frontal gyrus	9	48	8	34	146	.002	4.17
R. Inferior frontal gyrus	47	34	32	0	279	<.0001	5.11
L. Inferior frontal gyrus	44	−46	10	20	961	<.0001	4.45
**Temporal Lobe**							
L. Middle temporal gyrus	21	−50	−48	4	157	.002	4.17
L. Insula	13	−48	−22	20	101	.01	4.02
**Parietal Lobe**							
Postcentral gyrus	3	−38	−22	52	2095	<.0001	5.71
Superior parietal lobule	7	−30	−50	58	260	<.0001	4.22
**Occipital Lobe**							
R. Cuneus	17	8	−78	10	4909	<.0001	5.44
L. Middle occipital gyrus	18	−22	−92	16	572	<.0001	4.83
R. Middle occipital gyrus	18	26	−90	10	187	.001	4.09
**Cerebellum**							
L. Anterior culmen	N/A	−8	−28	−10	476	<.0001	4.68

*p*<.001, cluster level FDR corrected (T = 3.50); ET  = 10. N/A  =  Not applicable. L. =  Left hemisphere. R. =  Right hemisphere. Coordinates reported in Talairach space.

#### Medium > Slow

Comparing the medium to slow rates of sentence presentation, readers with dyslexia showed activations with peaks in right superior, middle, and inferior frontal gyri and left inferior frontal gyrus; left middle temporal gyrus and insula, superior parietal lobule and postcentral gyrus; bilateral middle occipital gyrus and right cuneus; and cerebellar regions (Table 3).

### Comparing Typical and Dyslexic Readers: Fast > Medium > Slow

Compared to readers with dyslexia on the parametric analysis (Fast > Medium > Slow), typical readers showed greater activation in a cluster with a peak in left middle frontal gyrus that extended into the inferior frontal gyrus, insula, and precentral gyrus; a cluster with a peak in left superior temporal gyrus that extended into the supramarginal gyrus, inferior parietal lobule, middle temporal gyrus, and cingulate cortex; and a cluster with a peak in the brainstem that extended into bilateral brainstem regions, medulla, and right cerebellum (Table 4; Figure 1, bottom panel). Readers with dyslexia did not show any activation greater than typical readers (Table 4).

**Table 4:** Activations for Typical Reader > Dyslexic Groups.

Region (peak activation)	BA	x	y	z	Cluster extent	*p*	Z score
***Typical_(Fast > Medium > Slow)_ > Dyslexic_(Fast > Medium > Slow)_***							
L. Middle frontal gyrus	9	−54	22	26	1319	<.0001	4.01
L. Superior temporal gyrus	22	−60	−46	16	324	.048	4.29
Cingulate gyrus	32	14	20	44	772	<.001	4.38
Brainstem/Cerebellum		−2	−32	−38	380	<.0001	4.01
***Dyslexic_(Fast > Medium > Slow)_ > Typical_(Fast > Medium > Slow)_***							
N/A							
***Typical_(Fast > Slow)_ > Dyslexic_(Medium > Slow)_***							
L. Superior frontal gyrus	6	−4	10	66	7165	<.0001	6.65
L. Inferior frontal gyrus	45	−44	24	6	38925	<.0001	6.39
***Dyslexic_(Medium > Slow)_ > Typical_(Fast > Slow)_***							
L. Anterior cingulate gyrus	24	−6	24	−4	2159	<.0001	4.45
Sub-lobar		34	−34	−6	470	.006	3.94
Sub-lobar		−22	−8	26	699	.001	3.88
Sub-lobar		32	−8	−4	324	.025	3.65

*p*<.01, cluster level FDR corrected (T = 2.51); ET  = 10. N/A  =  Not applicable. L. =  Left hemisphere.

**Figure 2:**
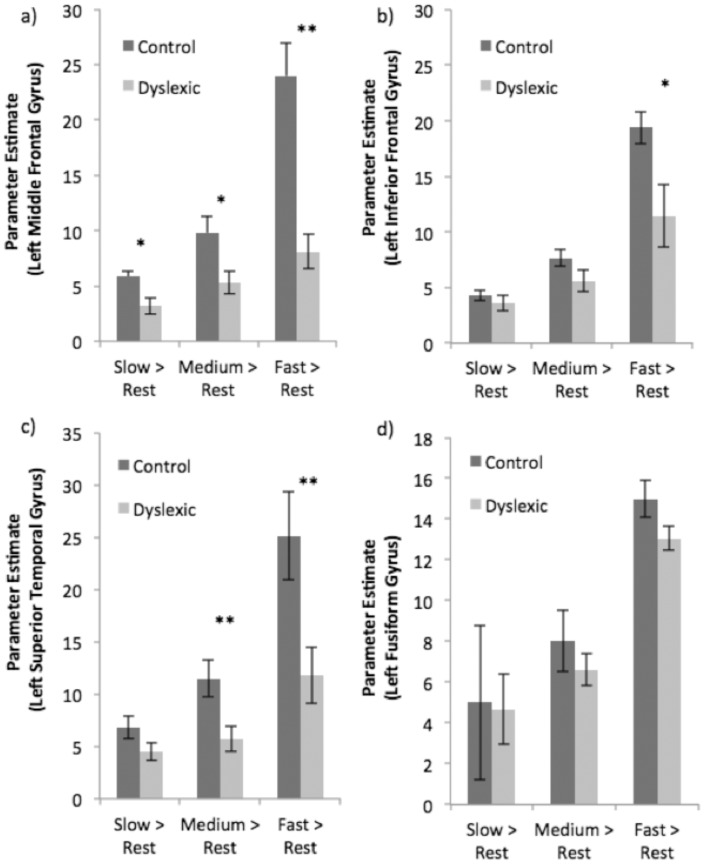
Comparison between Typical Reader and Dyslexic Groups (with standard error bars) showing region-of-interest activations for a) left middle frontal gyrus; b) left inferior frontal gyrus; c) left superior temporal gyrus; and d) left fusiform gyrus (visual word form area, VWFA). Note: *p<.05; **p<.01.

In order to characterize the nature of these group differences in activation, we extracted parameter estimate values for each reading rate, relative to the fixation baseline, from peaks of activation in regions showing greatest group differences (10 mm spheres around activation peaks in left middle (x = −54, y = 22, z = 26) and inferior (x = −44, y = 24, z = 6) frontal gyri and left superior temporal gyrus (x = −60, y = −46, z = 16)). In left middle frontal gyrus, typical readers showed significantly more activation than dyslexic readers at all rates (*p* = .01) (Figure 2a). In left inferior frontal (Figure 2b) and left superior temporal (Figure 2c) regions, there were no significant differences for the Slow condition (*p*>.05), but typical readers exhibited greater activation in the Medium [left middle frontal gyrus, *t*(22)  = 2.47, *p* = .02; left superior temporal gyrus, *t*(22)  = 2.73, *p* = .01] and Fast conditions [left middle frontal gyrus, *t*(22)  = 4.78, *p*<.0005; left superior temporal gyrus, *t*(22)  = 2.67, *p* = .02; left inferior frontal gyrus, *t*(22)  = 2.67, *p* = .02]. In addition, the putative visual word form area (VWFA; left fusiform gyrus) region of interest exhibited greater activation as a function of rate (*p*<.0001), but there was neither an effect of group nor a group x rate interaction (*p* = .41) (Figure 2d).

### Comparing conditions matched for in-scanner performance

**Figure 3:**
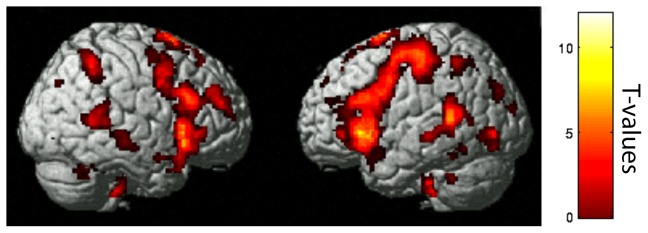
The Dyslexic Group showed reduced activation relative to the Typical Reader Group even when accuracy differences across conditions were equated between the groups. Greater activation for Fast > Slow contrast for Typical Readers versus Medium > Slow contrast for the Dyslexic Group (cluster level FDR corrected results displayed at p<.001). Color bar indicates T-values.

Typical readers (Fast > Slow) and readers with dyslexia (Medium > Slow) were compared based on matched in-scanner performance (Table 4). Typical readers showed significantly greater activation in almost all brain regions engaged by the task (Figure 3). Readers with dyslexia showed greater activations compared to typical readers in left anterior cingulate regions.

## Discussion

We compared the neural correlates of reading fluency in adult readers with and without dyslexia using an fMRI sentence reading paradigm that parametrically varied fluency demands by increasing the rate at which sentences were presented for semantic plausibility judgments. Faster presentation rates resulted in slower responses and reduced judgment accuracy in both groups. Readers with dyslexia were slower and less accurate across rates than typical readers, and their accuracy declined disproportionately as rates increased. In-scanner behavioral performance correlated with standardized measures of reading fluency, indicating that the scanner task explored the same underlying fluency processes, and also standardized measures of phonological awareness. For both typical and dyslexic readers, a large bilateral network of cortical, subcortical, and cerebellar systems supported fluent sentence reading. Readers with dyslexia showed less of an increase in activation, as a function of reading rate, in left prefrontal and left superior temporal cortices, anterior cingulate, and brainstem/cerebellar regions. The relationship between performance levels did not account for the differences in activation, because equating performance at different rates between the typical and dyslexic readers enhanced, rather than reduced, group differences. These findings point to brain regions that are associated with reading fluency in typical reading and with reading dysfluency in dyslexia.

### Defining and measuring fluent reading

The current study overcame several challenges in defining and measuring reading fluency directly using fMRI. Reading fluency was measured by using silent sentence reading in a word-by-word presentation format with the rate of word presentation manipulated on a sentence-by-sentence basis. Presentation rate was chosen as the independent variable because reading rate is a core aspect of reading fluency [56], and it can be manipulated in an fMRI task. Attention to the task was required as participants were asked to make semantic plausibility judgments following each sentence.

Silent reading of sentences was feasible for use in an fMRI study and similar to real world reading experiences. Implicit, or silent, word reading is effective in eliciting activations from brain areas associated with language processing [57]. Furthermore, for most adult readers, reading connected text silently is the most frequent interaction with written language, and predominantly involves decoding strings of words to extract meaning as opposed to reading isolated single words. Previous studies using word reading tasks have provided a basis for understanding the brain networks recruited for decoding and recognizing single words (i.e., isolated text), and studies using sentence reading tasks have identified brain regions recruited to process semantic or syntactic properties of sentences instantiated by relations among words. Sentence reading tasks can provide the additional advantage of localizing cognitive functions pertaining specifically to reading fluency - a dynamic aspect of reading behavior.

### Limitations

We presented sentences one word at a time to control reading rate. A limitation of this approach is that some sentence-reading processes that are typically engaged during the reading of text, such as the voluntary allocation of different viewing times for different words, or looking back at words, were not invoked in this design. Several behavioral observations indicate, however, that this task probed reading fluency processes. First, faster rates reduced judgment accuracy and latency across all participants. Second, the dyslexic group exhibited the expected deficits in accuracy and latency, and accuracy decreased disproportionately as a function of increasing reading rate. Third, when reading was examined across participants from typical and dyslexic groups, in-scanner performance correlated with scores from a standardized reading fluency test that is widely used in educational and clinical testing. These behavioral findings support the validity of the reading rate manipulation as a test of reading fluency.

### Brain regions associated with typical or impaired reading fluency

In typical readers, increased rates of word presentation likely influence many perceptual, phonological, semantic, syntactic, and pragmatic processes, and, correspondingly, resulted in increased activation in a large bilateral network of cortical, subcortical, and cerebellar regions. This activation pattern included brain regions implicated in processing visual (ventral occipital regions), phonological (inferior frontal gyrus, posterior superior temporal gyrus), and semantic (middle temporal gyrus, posterior superior temporal gyrus) information [58], [59].

The group with dyslexia showed many similarities to the typical group in regards to increased activation as a function of word presentation rate in a large bilateral network. The dyslexic group showed significantly less gain in activation relative to the control group in primarily left hemisphere regions, including left middle and inferior frontal gyri and left superior temporal gyrus. The left posterior superior temporal gyrus supports processing of semantic judgments, and shows greater activation as a function of greater semantic analysis [59]. The left inferior frontal gyrus has been implicated in semantic working memory [60], unrelated to general task difficulty [61], but related to competition between or selection among related semantic response options [62]. Although the current study did not directly investigate the distinct contributions of phonological and semantic processing, left inferior frontal gyrus activation in this study closely approximates the location identified in previous research (x = −37, y = 28, z = −9) showing greater activation for semantic versus phonological processing [63]. The left inferior frontal gyrus is also activated for extracting a coherent meaning from individual words in a sentence [64] and semantic processing of sentences [65]. At the same time, there is considerable overlap in brain regions associated with semantic and phonological analysis of language (e.g., the left posterior superior temporal gyrus) [58], [59]. To further characterize the relevant roles of the left inferior frontal gyrus and superior temporal gyrus in fluent reading, future analyses can compare activations for semantically appropriate and inappropriate sentences, or vary phonological demands.

Thus, some of the regions that showed significantly less activation in readers with dyslexia, which differed most at the fastest rate, are implicated in the control (left inferior frontal gyrus) or representation (left superior temporal gyrus) of semantic verbal knowledge. Weak responses in these regions associated with semantic processes during rapid or fluent reading could diminish comprehension during reading, as occurred for readers with dyslexia in the medium and fast conditions.

There was also greater activation in the group of typical readers in the cerebellum. Some studies have pointed to cerebellar anatomical differences in dyslexia [66], and it has been hypothesized that automaticity deficits in dyslexia may be associated with atypical cerebellar function [67]. The cerebellum, in addition to supplemental motor area (SMA) and primary motor cortex, shows increased recruitment for increasing rate (faster) and shorter duration when naming visually presented words [68], and for semantic and phonological processing [69]. The right cerebellar declive in particular has been implicated in automaticity in reading [70]. Functional connectivity between the cerebellum and inferior frontal and lateral temporal regions during reading suggests a coordinated cortico-cerebellar system that facilitates fluent reading [71]. The present findings are consistent with the impairment of the cerebellar component of this reading network in dyslexia.

Although activation was found in the purported visual word form area (VWFA) in the left temporo-occipital cortex and increased with reading rate, it did so similarly for both groups. This region has been associated with rapid processing of text for typical readers. Previously, this region has been found to show reduced activation in children with developmental dyslexia [72], [73]. Due to previous findings, we had expected to observe reduced activation in dyslexic adults as a function of reading rate. Perhaps the increasing intensity of visual processing across the faster rates of word presentation was such a strong manipulation that it dominated activation in the VWFA.

The most striking group differences occurred in left-hemisphere cortical regions implicated in semantic processing and required to perform the semantic analyses of the sentences. The present study, however, cannot determine what kinds of processing bottlenecks in the brain restricted the flow of information to brain regions involved in semantic analysis and judgment. Such bottlenecks may have been due to slow phonological decoding of single words, or impaired temporal processing that limited coordination of reading processes across words. The absence of a group difference in VWFA suggests the dyslexic group may not have been limited by the rate of orthographic processing per se, but only more targeted experiments can better elucidate the bases of the fluency impairment.

The activation differences between the groups could reveal the cause or the consequence of impaired fluency in dyslexia (or both). One approach towards this issue of interpretation is to compare typical and dyslexic groups under conditions where behavioral performance or comprehension is equated. This analytic approach was possible by comparing the difference between slow and medium rates in the dyslexic group to the difference between slow and fast rates in the typical reading group, because these comparisons did not show significant accuracy differences between the groups. When reading accuracies across conditions were equated, however, there remained large brain activation differences between the groups. Therefore, the activation differences between groups were not simply the consequence of worse performance by the group with dyslexia. Rather, weakened engagement of brain regions associated with semantic processing and automated reading may reflect the cause of the fluency deficits that make reading comprehension so challenging for many readers with dyslexia.
